# Mechanisms of Obesity-Induced Inflammation and Insulin Resistance: Insights into the Emerging Role of Nutritional Strategies

**DOI:** 10.3389/fendo.2013.00052

**Published:** 2013-05-10

**Authors:** Maeve A. McArdle, Orla M. Finucane, Ruth M. Connaughton, Aoibheann M. McMorrow, Helen M. Roche

**Affiliations:** ^1^Nutrigenomics Research Group, UCD Conway Institute, School of Public Health, Physiotherapy and Population Science, University College DublinDublin, Republic of Ireland

**Keywords:** obesity, inflammation, immune cell infiltration, insulin resistance, nutrient sensing, PUFA

## Abstract

Obesity and associated chronic inflammation initiate a state of insulin resistance (IR). The secretion of chemoattractants such as MCP-1 and MIF and of cytokines IL-6, TNF-α, and IL-1β, draw immune cells including dendritic cells, T cells, and macrophages into adipose tissue (AT). Dysfunctional AT lipid metabolism leads to increased circulating free fatty acids, initiating inflammatory signaling cascades in the population of infiltrating cells. A feedback loop of pro-inflammatory cytokines exacerbates this pathological state, driving further immune cell infiltration and cytokine secretion and disrupts the insulin signaling cascade. Disruption of normal AT function is causative of defects in hepatic and skeletal muscle glucose homeostasis, resulting in systemic IR and ultimately the development of type 2 diabetes. Pharmaceutical strategies that target the inflammatory milieu may have some potential; however there are a number of safety concerns surrounding such pharmaceutical approaches. Nutritional anti-inflammatory interventions could offer a more suitable long-term alternative; whilst they may be less potent than some pharmaceutical anti-inflammatory agents, this may be advantageous for long-term therapy. This review will investigate obese AT biology, initiation of the inflammatory, and insulin resistant environment; and the mechanisms through which dietary anti-inflammatory components/functional nutrients may be beneficial.

## Introduction

The global increase in body mass is an escalating societal concern. Concomitant environmental factors such as poor dietary habits, sedentary lifestyle, socioeconomic influences; and less frequently, genetic disorders that impact on hormone secretion and metabolism, result in weight gain. The world health organization (WHO) projects that by 2015, 2.3 billion adults will be overweight, body mass index (BMI) >25 (kg/m^2^) and more than 700 million will be obese BMI >30 (kg/m^2^). Consequently obesity-related co-morbidities including type 2 diabetes (T2D), cardiovascular disease (CVD), and non-alcoholic fatty liver disease (NAFLD) will continue to escalate (Kopelman, 2000; Mokdad et al., 2003).

**Figure 1:**
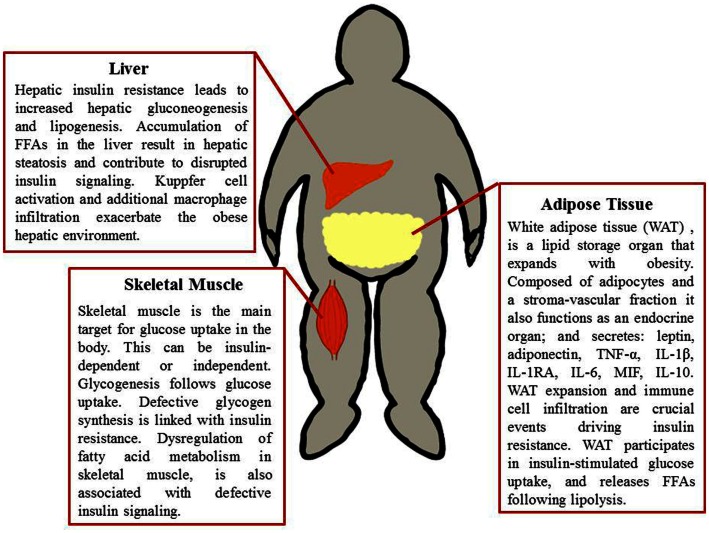
**Metabolic tissues implicated in obesity-induced insulin resistance**. Adipose tissue, liver, and skeletal muscle are involved in glucose uptake, glucose production, and glucose processing. These tissues therefore are paramount in obesity and the progression of insulin resistance. A combination of defective fatty acid storage and metabolism together with immune cell infiltration and a pro-inflammatory tissue milieu result in dysregulation of insulin signaling.

Substantial evidence indicates that obesity is linked to a state of chronic low-grade inflammation. Initially Hotamisligil et al. (1993) determined a link between obesity and inflammation; demonstrating that the pro-inflammatory cytokine tumor-necrosis factor (TNF)-α was expressed in adipose tissue (AT) of obese mice and linked to insulin resistance (IR). Significant advances in understanding the highly complex role of immuno-metabolism in health have since been accomplished. Consequently obesity is linked to pro-inflammatory cytokine secretion, immune cell infiltration, and disrupted function of tissues involved in glucose homeostasis, summarized in Figure 1. Dysfunctional lipid metabolism accompanies obesity and can impair insulin signaling; circulating free fatty acids (FFAs) have a negative effect on insulin target tissues, through the activation of inflammatory pathways, via cell surface pattern recognition receptors (PRRs) (Shi et al., 2006). Furthermore, accumulation of lipid derivatives, such as diacylglycerol (DAG) and ceramides can negatively regulate insulin action (Schenk et al., 2008).

The purpose of this review is to evaluate the current evidence in relation to obesity-induced IR, whereby the inflammatory axis plays a critical role in the progression and severity of IR. It is possible to attenuate the pro-inflammatory, insulin de-sensitizing milieu within the obesogenic environment. Weight management programs can attenuate T2D risk by reducing obesity and improving insulin sensitivity; however the long-term success of this approach is not optimal. Thus, are there effective nutritional strategies to attenuate the impact of inflammation mediated IR beyond weight loss?

## Adipose Tissue, Obesity-Induced Inflammation, and Insulin Resistance

There are two distinct forms of AT, brown adipose tissue (BAT) and white adipose tissue (WAT). BAT is typically associated with thermogenesis, although initially thought to disappear soon after birth in humans, evidence now suggests that BAT is present in adult humans in the supraclavical and paraspinal regions (Yoneshiro et al., 2011). Recent studies support the role of BAT functionality in adult humans relating to the control of body temperature and adiposity, which is of direct relevance to the metabolic syndrome (MetS) (Jacene et al., 2011; Ouellet et al., 2011; Nishio et al., 2012). BAT levels and activity are inversely correlated with BMI and conversely loss of BAT activity may be associated with the accumulation of WAT (Saito et al., 2009; Van Marken Lichtenbelt et al., 2009; Vijgen et al., 2011).

The depot most important to obesity and IR is WAT. WAT traditionally functions in lipid storage, storing triacylglycerides (TAG) following energy excess, and mobilizing these stores during periods of nutrient deprivation (Gregoire et al., 1998). With obesity and IR there is increased lipolysis, consequently there is an inappropriate spill over of TAG derived FFAs (Sethi and Vidal-Puig, 2007) and these FFAs can activate inflammatory pathways and impair insulin signaling. WAT also acts as an endocrine organ, releasing bioactive substances that have been coined adipokines, these include interleukin (IL)-6, IL-1β, TNF-α, leptin, and adiponectin. WAT is a complex multi-cellular organ, composed primarily of adipocytes. The stroma-vascular fraction (SVF) of AT contains several highly potent cells including adipocyte progenitor cells, or pre-adipocytes, and resident immune cell populations. In the IR state, pro-inflammatory cytokines activate several serine kinases, including IκB kinase (IKK) and JNK (Gual et al., 2005). These kinases have been shown to inhibit insulin action by promoting the phosphorylation of serine residues of the insulin signaling pathway, including serine phosphorylation of insulin receptor substrate-1 (IRS-1). In contrast with tyrosine phosphorylation of IRS-1 in the insulin sensitive state, serine phosphorylation impairs normal insulin signaling (Schenk et al., 2008).

### Adipocytes

**Figure 2:**
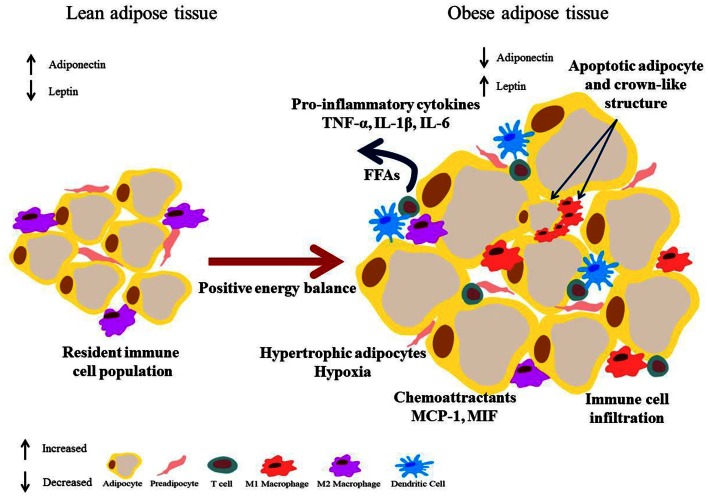
**Obese adipose tissue expansion – resultant inflammation and metabolic dysregulation**. Excess energy leads to adipose expansion with hypertrophic adipocytes that secrete chemoattractants such as MCP-1, drawing immune cells into the tissue. Secretion of pro-inflammatory mediators such as TNF-α, IL-1β, and IL-6 by adipocytes, pre-adipocytes, and infiltrating immune cells results in polarization of macrophages to a pro-inflammatory M1 phenotype, and drive an inflammatory T cell population. Augmented lipolysis leads to increased levels of FFAs. This environment negatively impacts on the insulin signaling pathway and a state of insulin resistance results. Additionally hypertrophic adipocytes are also linked with hypoxia.

White adipose tissue is unique in its plasticity, it can adapt quickly to nutrient deprivation and hyper-nutrition alike. The flexibility of WAT is largely due to the hypertrophic and hyperplastic changes in adipocytes. WAT plasticity has an important role in determining metabolic health (Virtue and Vidal-Puig, 2008). The expansion of WAT that occurs with weight gain is accompanied by changes in cytokine and chemokine secretion, hypoxia, cell death, immune cell infiltration, and dysregulation of fatty acid (FA) metabolism and storage, see Figure 2. Adipocyte hypertrophy modulates the adipose secretome (Osborn and Olefsky, 2012). Mature adipocytes secrete IL-6, monocyte chemoattractant protein (MCP)-1, leptin, and adiponectin; which can act in an autocrine, paracrine, or endocrine manner to regulate lipid and glucose homeostasis (Curat et al., 2004). Expression of inducible nitric oxide synthase (iNOS), MCP-1 and IL-6 are concomitantly increased in the mature adipocyte fraction of obese mice, in parallel with increased 11β-hydroxysteroid dehydrogenase type I (HSD1) levels (Ishii-Yonemoto et al., 2010). In *in vitro* cultures TNF-α, IL-1β, and IL-6 up-regulate 11β-HSD1 (Tomlinson et al., 2001). Increased MCP-1 expression promotes monocyte infiltration of the WAT, these then differentiate into adipose tissue macrophages (ATM). Adipocytes also induce the expression of the adhesion molecules ICAM-1 and PECAM-1 on endothelial cells (Curat et al., 2004) which further attract monocytes to the region. ATM secrete additional chemokines and cytokines, further exacerbating the pro-inflammatory environment (Osborn and Olefsky, 2012).

### Stroma-vascular fraction

**Figure 3:**
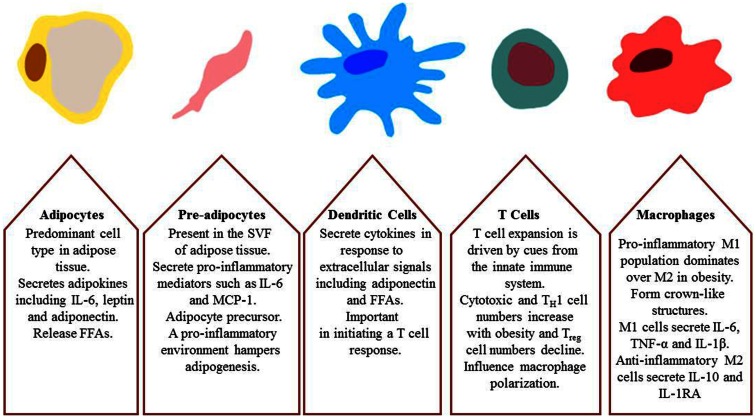
**Major cell types involved in obesity-induced inflammation and insulin resistance; adipocytes, pre-adipocytes, dendritic cells, T cells, and macrophages**.

The SVF of WAT is composed of several metabolically active and inflammatory cells, summarized in Figure 3; including pre-adipocytes, fibroblasts, endothelial cells, dendritic cells (DCs), T cells, mast cells, granulocytes, and macrophages (Calder et al., 2011) embedded in an extra cellular matrix. This fraction plays a critical role in healthy fat pad expansion (Sun et al., 2011). In response to a high-fat diet (HFD) and obesity, there is an increase in SVF cell number, the phenotype of which adversely affects metabolism (Strissel et al., 2010).

### Pre-adipocytes

Two distinct adipocyte cell types were identified in human omental fat (Julien et al., 1989). Mature adipocytes with lipid droplets were observed in close proximity to much smaller nucleated cells containing less lipid, referred to as pre-adipocytes. Increasing body weight is associated with increased numbers of pre-adipocytes and it has been suggested that the expansion of this cellular population is associated with IR (McLaughlin et al., 2007). There is much speculation as to the origins of pre-adipocytes (Cawthorn et al., 2012). Hollenberg and Vost (1969) utilized tritiated thymidine incorporation in WAT and identified the SVF as the source of new adipocyte formation. Adipocyte stem cells (ASCs) are likely mesenchymal stem cells that reside in the WAT and give rise to adipocytes; with pre-adipocytes representing an intermediary stage in this process (Zuk et al., 2001; Cawthorn et al., 2012).

Cytokines have potent effects on pre-adipocyte biology. TNF-α, interferon (IFN)-γ, IL-1β, and IL-6 impair adipogenesis and lipid accumulation in 3T3-L1 pre-adipocytes (Gustafson and Smith, 2006; McGillicuddy et al., 2009). Specifically, IL-6 treatment reduced adiponectin, resistin, glucose transporter type (GLUT)-4, and IRS-1 expression; while TNF-α treatment increased secretion of IL-6 and MCP-1 from pre-adipocytes (Chung et al., 2006). Indeed pre-adipocytes secrete greater levels of pro-inflammatory mediators such as IL-6 and MCP-1 than adipocytes (Poulain-Godefroy and Froguel, 2007; Mack et al., 2009). Phospholipase Cδ1 (PLCδ1) has also been shown to regulate 3T3-L1 pre-adipocyte differentiation (Hirata et al., 2011), this enzyme regulates adipogenesis. Pre-adipocytes secrete basic fibroblast growth factor (bFGF/FGF-2), involved in promoting vascular endothelial cell growth, levels of which are increased with obesity (Bell et al., 2006; Sun et al., 2011). Basic fibroblast growth factor is involved in recruiting monocytes and neutrophils under chronic inflammatory conditions, it acts synergistically with TNF-α and IFN-γ and in the absence of these cytokines bFGF could not induce leukocytes recruitment (Zittermann and Issekutz, 2006).

Pre-adipocyte factor-1 (Pref-1) is a transmembrane protein expressed exclusively by pre-adipocytes and inhibits adipogenesis (Chung et al., 2006; O’Connell et al., 2011). Pref-1 levels are increased in metabolically unhealthy WAT (O’Connell et al., 2011) and levels correlate with ATM number. The capacity of pre-adipocytes to undergo adipogenesis is a critical factor in obesity and IR. Adipogenic potential is impeded with age and by various cytokines, via the transcription factors CCAAT/enhancer binding protein (C/EBP)1α (Karagiannides et al., 2001) and peroxisome proliferator-activated receptors (PPAR)-γ (Hotta et al., 1999). Reduced capacity for *de novo* adipogenesis, coupled with increased FFA storage demand in obesity may account for the switch from hyperplastic to hypertrophic WAT, which in turn has negative implications in terms of impeding FFA storage capacity and leakage.

### Adipose tissue macrophages

Adipose tissue macrophages can be classified based on their surface marker expression and/or their chemokine and cytokine secretion profile. ATM number and phenotype is altered in genetic and diet induced obesity. In the obese state there are greater proportions of classically activated M1 but less M2 macrophages; which would otherwise play a role in the resolution of inflammation (Lumeng et al., 2007; Fujisaka et al., 2009). M1 macrophages secrete pro-inflammatory cytokines TNF-α, IL-1β, and IL-6; whereas alternatively activated M2 macrophages secrete anti-inflammatory cytokines including IL-10 and IL-1 receptor antagonist (RA) (Lumeng et al., 2007). The degree of ATM infiltration is associated with the progression of IR (Osborn and Olefsky, 2012). It is likely that M1 ATMs originate from monocytes in circulation rather than originating in the SVF (Calder et al., 2011; Oh et al., 2012).

The immuno-phenotype of macrophages is highly plastic in response to their surrounding milieu (Mosser and Edwards, 2008). As obesity progresses the ATM phenotype switches from anti-inflammatory M2 to the pro-inflammatory M1 type, through a dynamic process spanning a spectrum from M1 to M2 states (Lumeng et al., 2007; Osborn and Olefsky, 2012). ATM cluster around necrotic adipocytes in arrangements coined crown-like structures (CLS). M1 ATM infiltration decreases insulin sensitivity as a result of greater TNF-α, IL-1β, and IL-6 secretion, which induce an insulin resistant environment. FFAs can activate macrophages *in vitro* acting through Toll like receptor (TLR)2 and TLR4 to induce MCP-1, IL-6, and IL-1β expression (Nguyen et al., 2007). FFAs also induce PAI-1 secretion from macrophages *in vitro*; however this induction occurred only in the presence of 3T3-L1 adipocytes in the culture environment, suggesting FFA’s and adipocyte secretions act synergistically to influence macrophage secretions (Kishore et al., 2010). ATM infiltration can be detected after only 1 week of HFD feeding (Lynch et al., 2012) and increase progressively with time and in proportion to the degree of obesity. With obesity ATM also take on a foam cell-like role, accumulating excess lipid (Prieur et al., 2011). Weight loss is associated with lower ATM number (Kosteli et al., 2010) however initial rapid weight loss is also accompanied by transient infiltration of macrophages (Granneman et al., 2005; Sun et al., 2011).

There is some uncertainty when distinguishing macrophages from DCs (Geissmann et al., 2010); these cell types overlap in terms of function and molecular characterization. Both are phagocytic antigen presenting cells (APC) and also share certain cell surface markers including CD11c, MHC II, and F4/80. The Immunological Genome Project has generated detailed gene expression profiles and regulatory pathways that enable discrimination between macrophages and DCs and additionally to distinguish between different populations of these cell types (Gautier et al., 2012). This project shows that there was great diversity between different macrophage populations but detected only 39 mRNA transcripts common to all macrophage types but not to DCs.

### T cells

Adipose tissue macrophages were the first immune cells associated with IR, however in terms of WAT infiltration they are preceded by other immune cells including T cells. Different T cell subsets are involved in obesity and WAT infiltration. Regulatory T cells (T_reg_) normally constitute 5–20% of the CD4^+^ T cell population and play a crucial role in maintaining immune homeostasis (Feuerer et al., 2009). T_reg_ secrete anti-inflammatory cytokines, inhibiting macrophage migration, and promoting an M2 macrophage phenotype (Osborn and Olefsky, 2012). A decline in T_regs_ accompanies increasing weight gain in both mouse and human (Feuerer et al., 2009; Winer et al., 2009a). The T_reg_ depleted mouse has lower insulin-stimulated insulin receptor tyrosine phosphorylation in both epididymal fat and liver, accompanied by reduced AKT phosphorylation (Feuerer et al., 2009). Anti-inflammatory CD4+ T helper (T_H_) 2 cells secrete IL-4 and IL-13 (Winer et al., 2009a). Interleukin-4 induces M2 macrophages that secrete IL-10, which in turn is proposed to have insulin sensitizing potential.

Pro-inflammatory T_H_1 cells secrete IFN-γ that drives polarization of pro-inflammatory macrophages, that in turn secrete IL-1, IL-6, and TNF-α (Winer et al., 2009a). T_H_1 cells can activate macrophages (Zhu and Paul, 2008). HFD feeding increased T_H_1 cell number thus negating the anti-inflammatory secretions from T_reg_ and T_H_2 cells (Winer et al., 2009a). Interleukin-6 in conjunction with IL-23, TGF-β, and IL-1, drive the proliferation of CD4^+^ T_H_17 cells (Kikly et al., 2006; Brereton et al., 2009). Activation of ERK in DCs is crucial in driving IL-1β and IL-23 production that further drive the expansion of T_H_17 cells (Brereton et al., 2009). T_H_17 cells secrete the pro-inflammatory cytokines IL-17A and IL-17F and also TNF-α, IL-6, GM-CSF, CXCL1, and CCL20. Winer et al. (2009b) reported an increase in T_H_17 cells in the spleens of HFD-fed mice, but T_H_17 cells were absent in HFD-fed IL-6^−/−^ mice. HFD-fed Rag1^−/−^ mice reconstituted with CD4^+^ T cells had lower weight gain than those reconstituted with CD8^+^ T cells, both had similar food intake (Winer et al., 2009a). The CD4^+^ T cell recipient mice also had smaller adipocytes in both subcutaneous and epididymal fat pads. The infiltration of CD8^+^ T cells to WAT is an early event in obesity, whereby obese WAT can activate CD8^+^ T cells that subsequently recruit macrophages (Nishimura et al., 2009; Harford et al., 2011). The percentage of CD8^+^CD4^−^ T cells in the SVF fraction of epididymal fat increased in HFD-fed mice after just 2 weeks. CD8^+^CD4^−^ T cell number increased for up to 11 weeks on HFD while both CD8^−^CD4^+^ and T_reg_ numbers decreased over time (Nishimura et al., 2009). Depletion of CD8^+^ cells resulted in a significant reduction in CD8^+^CD4^−^ T cells numbers in epididymal fat pads, and reduced M1 macrophage infiltration; without altering the M2 fraction in HFD-fed mice. In order to demonstrate the effects of obese versus lean WAT on CD8^+^ T cells, epididymal fat pads from chow and HFD-fed mice were co-cultured with splenic CD8^+^ T cells; the obese WAT induced T cell proliferation to a greater extent than the lean WAT (Nishimura et al., 2009).

Invariant natural killer T (iNKT) cells are a population of innate T cells present in WAT; obesity is accompanied by a decline in iNKT cells in WAT (Lynch et al., 2012). This cell type can recognize lipid antigens on MHC-like glycoprotein CD1 (Brigl and Brenner, 2004; Lynch et al., 2009b). DCs express CD1 and *in vivo* may activate iNKT cells (Exley and Koziel, 2004). There is uncertainty about naturally occurring ligands recognized by iNKT cells; endogenous lysosomal glycosphingolipid and isoglobotrihexosylceramide in addition to bacterial glycosphingolipids are proposed iNKT ligands (Zhou et al., 2004; Kinjo et al., 2005; Lynch et al., 2009b). iNKT cells are found at a higher frequency in mouse than in human, however they are particularly present in the human omentum, this region also contains the highest concentration of CD1d^+^ cells (Lynch et al., 2009b). iNKT cells comprise up to 50% of resident T cells in the liver (Eberl et al., 1999) and in the WAT they produce T_H_2 type cytokines. Jα18 deficient mice and CD1d^−/−^ mice lack iNKT cells, with this absence there is an increase in macrophage number in WAT (Lynch et al., 2012). Both show greater M1 infiltration of WAT following HFD feeding. CD1d^−/−^ mice have decreased adiponectin and increased leptin levels in WAT and plasma (Schipper et al., 2012). Lean mice deficient in iNKT develop an insulin resistant phenotype in the absence of WAT inflammation (Schipper et al., 2012). These lean mice also displayed adipocyte hypertrophy but there was no change in epididymal fat pad weight relative to wild-type mice.

### Dendritic cells

Dendritic cells are considered the principal APC of the immune system and are crucial for initiating a T cell response (Sallusto and Lanzavecchia, 1994). They process phagocytosed particles and use peptide information from these to present to T cells via MHC class I and class II molecules. DCs are the only cell type that can induce naïve T cell differentiation (Sallusto and Lanzavecchia, 1994; Bhattacharya et al., 2011). Mature DCs secrete cytokines that influence different cell responses, e.g., naïve T cells to T_H_1, -2, -17, T_regs_ and therefore DCs link innate and adaptive arms of the immune system. T_H_1 cell induction follows pro-inflammatory IL-12p70 secretion by DCs (McGuirk et al., 2005); T_H_1 cells then produce IFN-γ and can prevent the development of T_reg_ cells (DePaolo et al., 2011). Additionally IFN-γ feeds back to DCs ensuring the continued production of IL-12p70. Inactivation of MEK1/2 in the MAPK pathway leads to a reduction in IL-1β and IL-23 production but does not result in diminished IL-12p70 production.

Adipose and bone marrow DC number increases following HFD feeding in mice (Reynolds et al., 2012). Further studies have shown HFD feeding also results in DC infiltration of the liver in addition to the WAT (Stefanovic-Racic et al., 2012). DCs present in the liver of obese mice have increased CD86 expression, suggesting that the obese environment promotes DC maturation. Additionally Flt3^−/−^ mice, lacking DCs, showed reduced macrophage infiltration of liver and WAT. DCs isolated from the SVF of lean and obese mice contained different population subsets (Bertola et al., 2012); CDllc^high^/F4/80^low^ DCs from obese mice induced the differentiation of T_H_17 cells that produced high levels of IL-17, whereas CDllc^high^/F4/80^neg^ DCs induced both T_H_1 and T_H_17 cells that produced low levels of their respective cytokines IFN-γ and IL-17. In lean mice CDllc^high^/F4/80^neg^ DCs only induced T_H_1 cells and CDllc^high^/F4/80^low^ DCs initiated a weak T_H_17 cell expansion.

Zhong et al. (2013) investigated the role of dipeptidyl peptidase-4 (DDP4/CD26) in human DCs in relation to T cell activation. Dipeptidyl peptidase-4 was first identified as an enzyme involved in the degradation of glucagon-like peptide (GLP)-1 (Yazbeck et al., 2009). Dipeptidyl peptidase-4 expression was investigated in human peripheral blood and the SVF fraction from the omentum of healthy lean subjects (Zhong et al., 2013). Expression of DPP4 was greater on DCs and macrophages from the SVF fraction than those from the peripheral blood. Dipeptidyl peptidase-4 expressing macrophages were measured from visceral adipose tissue (VAT) of obese subjects and levels were higher than observed in lean controls; both HFD-fed mice and *ob/ob* mice also showed increase DPP4 expression in WAT, and on DCs and macrophages (Zhong et al., 2013).

### Natural killer cells

Natural killer (NK) cells represent the first line of immune defense (Shi et al., 2000). They produce IFN-γ and can drive T_H_1 cell expansion (Yadav et al., 2011), they also produce IL-17 (Cua and Tato, 2010). Subjects deemed obese yet metabolically healthy had a greater number of CD8^+^ cells and NK cells than their “unhealthy” counterparts, but both groups had fewer circulating NK cells than lean subjects (Lynch et al., 2009a). NK cells differed in phenotype between the two groups of obese patients; there was increased expression of inhibitory markers such as NKB1 and CD158b in the unhealthy group. Adipokines can influence NK cells and leptin was shown to have an inhibitory effect on NK cells in lean but not obese subjects while adiponectin enhanced NK activity in obese subjects (O’Shea et al., 2010).

### Eosinophils

Eosinophils are classically associated with allergy but are also associated with M2 macrophages. Eosinophils are present in the WAT of mice and produce IL-4 thus act to replenish M2 macrophages; they account for 4–5% of SVF cells in the WAT (Wu et al., 2011). T_H_2 cell IL-4 production may complement eosinophil IL-4 secretion, or indeed make it redundant (Maizels and Allen, 2011). A decline in eosinophil numbers has been reported in HFD-fed mice (Wu et al., 2011) and in the absence of eosinophils M2 macrophage numbers become diminished.

## The Liver and Skeletal Muscle are Critical Organs Adversely Affected in Obesity-Induced Insulin Resistance

### Liver

Obesity induces morphological and metabolic alterations in other peripheral tissues including the liver and skeletal muscle. NAFLD is a complex metabolic disorder transpiring to be one of the most prevalent liver conditions in the western world (Flegal et al., 2002; Utzschneider and Kahn, 2006). NAFLD is strongly linked with obesity, IR, and T2D (Seppala-Lindroos et al., 2002; Adams et al., 2005). This condition represents multiple pathogenic states from steatosis to steatohepatitis which can progress to cirrhosis and liver failure (Farrell and Larter, 2006). In an obese state the liver exhibits selective IR; the inhibitory effect of insulin on hepatic gluconeogenesis is interrupted while its action on *de novo* lipogenesis is enhanced, resulting in chronic hyperglycemia and hypertriglyceridemia (Schwarz et al., 2003; Brown and Goldstein, 2008). The mechanisms responsible for hepatic IR are controversial and causality has been attributed to several factors.

The “portal hypothesis” suggests enhanced visceral tissue lipolysis, resultant from obesity-induced IR, exacerbates FFA influx into the liver via the portal vein, contributing to hepatic steatosis (Bjorntorp, 1990; Berraondo and Martínez, 2000; Utzschneider and Kahn, 2006). This increase in hepatic FFA may induce hepatic IR by promoting protein kinase C (PKC)δ translocation from the cytosol to the membrane, leading to impaired IRS-associated phosphatidylinositol (PI)3-kinase activity (Lam et al., 2002). Additionally elevated WAT derived pro-inflammatory cytokine secretion including TNF-α and reduced adiponectin secretion have also been implicated in hepatic IR (Berg et al., 2001). Alternatively excessive TAG accumulation within the liver has been implicated in hepatic IR (Utzschneider and Kahn, 2006). Patients with NAFLD display elevated *de novo* lipogenesis, which reflects the inability for insulin to impede lipogenesis (Donnelly et al., 2005). NAFLD associated hyperinsulinemia can stimulate lipogenic transcription factor sterol receptor binding protein (SREBP)-1c, and inhibit fork head transcription factor Foxa2 activity, promoting enhanced FA synthesis and reduced β-oxidation (Wolfrum et al., 2004; Tamura and Shimomura, 2005; Gonzalez-Baro et al., 2007). Furthermore, HFD-fed mice have elevated hepatocyte ceramide secretion (Boon et al., 2013), this secretion profile is linked to plasma ceramide levels suggesting that the liver is the principal producer of circulating ceramide.

The liver has a large population of resident macrophages or Kupffer cells (KC) (Neyrinck et al., 2009). NAFLD is linked with the inflammatory activation of KCs (Odegaard et al., 2008). Increased adiposity activates KC, without altering total KC number (Cai et al., 2005). Activation of KC is associated with hepatic IR (Neyrinck et al., 2009; Lanthier et al., 2010) and the production of inflammatory mediators including TNF-α and reactive oxygen species (ROS). Similar to ATM, KC present immense plasticity and can alter between a pro-inflammatory M1 and anti-inflammatory M2 phenotype, in response to their specific environment (Gordon, 2003). Ablation of PPAR-δ, an important FA sensor in hematopoietic cells, polarized KC to an M1 activation state augmenting HFD-induced hepatic steatosis through a reduction in hepatic β-oxidation (Odegaard et al., 2008). HFD fed mice depleted of KCs, have decreased lipogenic gene expression and suppression of hepatic glucose 6-phosphatase (G6Pase), a crucial gene involved in gluconeogenesis (Neyrinck et al., 2009). The G protein-coupled receptor (GPCR) GPR105 is involved in additional macrophage infiltration of the liver and the ensuing inflammatory and insulin resistant state (Xu et al., 2012).

### Skeletal muscle

Skeletal muscle is the main target organ for glucose uptake in the body, responsible for 80% of glucose disposal in man (Lorenzo et al., 2008; DeFronzo and Tripathy, 2009). Glycogen synthesis is the principal pathway for glucose disposal in both normal and T2D subjects (Shulman et al., 1990). Defective glycogen synthesis has a causative role in IR and T2D (Shulman, 2000). Insulin stimulation in T2D does not increase glucose-6-phosphate, an intermediary metabolite of glucose transport and glycogen synthesis. This suggests that T2D is associated with either decreased glucose transport activity or decreased hexokinase II activity. Further investigation elucidated that glucose transport is the rate-controlling step in insulin-stimulated glycogen synthesis (Cline et al., 1999).

Increased circulating FFAs and dysregulation of intramyocellular FA metabolism can result in a ∼50% decrease in insulin-stimulated glycogen synthesis in muscle (Roden et al., 1996). This is a consequence of reduced glucose-transport activity and defective PI3K activity (Dresner et al., 1999; Petersen and Shulman, 2002). *In vivo* infusion of a lipid emulsion in rats resulted in an increase in intracellular C18:2 CoA and DAG in skeletal muscle (Yu et al., 2002). Additionally reduced tyrosine phosphorylation of IRS-1 and reduced IRS-1 associated PI3K activity followed lipid infusion; this coincided with increased PKCθ activation. Combined these changes result in decreased insulin-stimulated glucose transport activity. Circulating ceramides are increased with T2D, in particular elevated LDL-ceramide is associated with IR (Boon et al., 2013). Infusion of LDL-ceramide into lean mice resulted in reduced glucose disposal in skeletal muscle and decreased phosphorylation of AKT (Boon et al., 2013). In skeletal muscle, IKKβ signaling increased coincident with increased expression of NF-κB target genes TNF-α, IL-6, and IL-1β. LDL-ceramide also decreased insulin-stimulated glucose uptake through decreased GLUT4 translocation.

Interleukin-6 levels are elevated in obesity, likely due to greater WAT IL-6 secretion; and are associated with IR and T2D risk (Spranger et al., 2003; Pedersen and Febbraio, 2008). The functional role and consequence of IL-6 in skeletal muscle is highly complex and potentially counterintuitive. In its role as a myokine, IL-6 expression is enhanced in contracting skeletal muscle and released after exercise, when insulin sensitivity is enhanced (Ostrowski et al., 1998; Steensberg et al., 2000). Skeletal muscle metabolism is enhanced by IL-6, increasing AMP-activated protein kinase (AMPK) α2 activity, FA oxidation, and glucose uptake (Kelly et al., 2009). Interleukin-6 signaling is abnormal in obese and T2D subjects muscle precursor cells. There is reduced skeletal muscle IL-6 receptor expression in obesity and abnormal STAT3/suppressor of cytokine signaling 3 (SOCS3) signaling, and attenuated IL-6 induced AMPKα2 activation with T2D (Scheele et al., 2012).

Hong et al. (2009) investigated IL-10 action in skeletal muscle glucose homeostasis; using a transgenic mouse model with muscle specific overexpression of IL-10. Following HFD feeding, IL-10 over-expressing mice had improved insulin sensitivity relative to HFD-fed control mice; additionally increased tyrosine phosphorylation of IRS-1 was demonstrated. There was reduced macrophage infiltration of skeletal muscle in the HFD transgenic mice and a corresponding reduction in IL-6 and TNF-α secretion in the muscle. TNF-α expression has been shown to be increased in muscle biopsies from IR subjects (Saghizadeh et al., 1996).

Insulin-independent glucose transport in skeletal muscle occurs following AMPK activation (Fujii et al., 2006). Leptin increases fatty oxidation and decreases fat storage in muscle via AMPK activation. HFD fed mice show decreased leptin-stimulated AMPK activation (Martin et al., 2006), due to abrogated acetyl-CoA carboxylase (ACC) activity downstream of the AMPK pathway. SOCS3, a regulator of leptin, is elevated in the skeletal muscle of HFD-fed mice (Yang et al., 2012). Overexpression of SOCS3 impairs leptin-stimulated AMPK activation, reduced tyrosine phosphorylation of IRS-1, PI-3-kinase activity, and AKT phosphorylation (Yang et al., 2012).

White adipose tissue derivatives, either FA or cytokine/inflammatory derived have a plethora of adverse and synergistic effects on hepatic and skeletal metabolism, that further augment the impact of dysregulated WAT metabolism in obesity.

## Inflammatory Mediators Involved in Obesity-Induced Insulin Resistance

Metabolic dysregulation has been attributed to numerous pro-inflammatory cytokines secreted by the altered immune cell milieu of obese WAT, including IL-1, TNF-α, MIF, and IL-6, all of which have been documented in disrupting insulin signaling.

### Interleukin-1 family

The IL-1 superfamily includes IL-1α, IL-1β, IL-1RA, IL-18, IL-33, and IL-37 (Dinarello, 2009; Akdis et al., 2011). Interleukin-1 is a multifunctional pro-inflammatory cytokine, produced by numerous innate immune cells including monocytes/macrophage and DCs (Garcia et al., 2006; Tilg and Moschen, 2008). Interleukin-1α and IL-1β mediate their actions through the IL-1 receptor 1(IL-1R1) and potently induce the production of inflammatory cytokines including IL-6. Conversely IL-1α and IL-1β differ in their maturation and secretion, IL-1α is secreted in a biologically active state while IL-1β activation is dependent on the cleavage of pro-IL-1β to mature IL-1β by caspase-1 (Dinarello et al., 2010). Interleukin-1β disrupts adipogenesis in human and murine cell models (Lagathu et al., 2006). IL-1 signaling cascade involves the activation of NF-κB and JNK MAPK pathways (Stylianou and Saklatvala, 1998). In contrast, anti-inflammatory IL-1RA binds to the IL-1R1 and blocks signaling events due to its lack of an essential IL-1R accessory protein (Tack et al., 2012). In recent years the full importance of IL-1 signaling in obesity, adipocyte dysfunction, and IR has been recognized.

Increased circulating IL-1β concentrations are associated with greater risk of developing T2D (Spranger et al., 2003). Obese VAT expresses more IL-1β and IL-1R1 (Juge-Aubry et al., 2003). Interleukin-1α impairs insulin-stimulated tyrosine phosphorylation of IRS-1 in 3T3-L1-adipocytes (He et al., 2006). Furthermore IL-1β inhibited insulin-stimulated glucose uptake into 3T3-L1-adipocytes, an effect proposed to be mediated via IL-1β induced ERK activation (Jager et al., 2007). Correspondingly lack of IL-1R1 protects mice from HFD-induced glucose intolerance (De Roos et al., 2009; McGillicuddy et al., 2011). Treatment of HFD-fed mice with IL-1RA ameliorated glucose intolerance and protected against obesity-induced pancreatic β-cell destruction (Sauter et al., 2008). However a certain element of controversy still surrounds IL-1 regarding its function *in vivo*. Interleukin-1RA^−/−^ mice exhibit a lean phenotype concomitant with enhanced energy expenditure and improved insulin sensitivity (Matsuki et al., 2003; Isoda et al., 2005). On the other hand IL-1R1^−/−^ mice develop mature onset obesity with reduced glucose homeostasis (Garcia et al., 2006). Together these studies highlight the pathological significance of IL-1 to obesity associated metabolic dysregulation but also highlight the complexity of this signaling molecule.

### Processing active IL-1β via NLRP3 inflammasome

The processing and activation of IL-1β *in vivo* comprises a multifaceted network of events wherein two individual stress response signals are required to “prime” and “activate” IL-1β. Activation of TLR4 via stress signals such as lipopolysaccharide (LPS) or saturated fatty acids (SFA) delivers the first “hit” necessary for production of pro-IL-1β (Wen et al., 2011). The subsequent processing of pro-IL-1β to mature biologically active IL-1β is dependent on the NOD like Receptor (NLRP)3-caspase 1 inflammasome complex (Mills and Dunne, 2009). The NLRP3 inflammasome is a multi-molecular complex recently demonstrated as central to obesity-induced IR. This complex is comprised of NLRP3, the adaptor molecule-apoptosis-associated speck-like protein containing a CARD and pro-caspase-1. Assembly of the NLRP3 inflammasome is mediated through a diverse range of endogenous and exogenous stressors including FFAs, glucose, adenosine triphosphate (ATP), uric acid, and ROS (Netea et al., 2009). Activation of the inflammasome induces caspase-1 activity required for cleaving pro-IL-1β to mature IL-1β. Indeed the importance of the NLRP3 inflammasome and caspase-1 activity in obesity-induced IR has been recently highlighted. Vandanmagsar et al. confirmed that weight loss through calorie restriction or exercise diminished NLRP3 expression in WAT. Moreover NLRP3^−/−^ mice were protected from the adverse metabolic effects elicited by HFD; demonstrated by improved glucose homeostasis, greater insulin-induced pAKT activity in VAT, subcutaneous adipose tissue (SAT), liver and muscle. Interestingly, similar to IL-1R1 deficient mice, lack of NLRP3 did not alter the ATM M1/M2 ratio (Vandanmagsar et al., 2011). Stienstra et al. illustrated that caspase-1 expression was amplified during adipocyte differentiation and this increase was associated with reduced insulin sensitivity. Lack of caspase-1 or NLRP3 increased adipogenesis and lipid accumulation in adipocytes. Furthermore, lack of caspase-1 prevented HFD-induced IR (Stienstra et al., 2010). These studies indicate that the inflammasome which modulates IL-1 activation/mediated inflammation is a critical regulator of inflammation and WAT function.

### Tumor-necrosis factor-α

Tumor-necrosis factor-α is a potent pro-inflammatory cytokine, primarily secreted from monocytes and macrophages, via the activation of MAPK and NF-κB signaling pathways, resulting in the release of other inflammatory cytokines such as IL-1β and IL-6 (Chen and Goeddel, 2002; De Luca and Olefsky, 2006). It was the first inflammatory mediator linked with obesity-induced IR (Hotamisligil et al., 1993). Chronic treatment of 3T3-L1 adipocytes with TNF-α activated intracellular IKKβ and reduced tyrosine phosphorylation of IRS-1, ultimately leading to impaired insulin action. Obese rodents administered TNF-α neutralizing antibody exhibited reduced hyperinsulinemia (Hotamisligil et al., 1994). Whole body deletion of TNF-α or its corresponding receptor TNF receptor 1 (TNFR1) gene partially protects mice from obesity-induced IR (Uysal et al., 1997). Several studies have shown TNF-α can also indirectly alter insulin sensitivity. The treatment of 3T3-L1 adipocytes with TNF-α increased lipolysis but also down-regulated adipogenic genes; PPAR-γ and C/EBP. Furthermore TNF-α activated NF-κB suppressed genes involved in lipid uptake and storage (Ruan et al., 2002). Obese human subjects exhibit elevated circulating levels of TNF-α (Hotamisligil et al., 1995; Kern et al., 1995). However the benefits of anti-TNF therapy in T2D are limited.

### Interleukin-10

Interleukin-10 is an anti-inflammatory cytokine produced by monocytes, M2 ATMs, DCs, T cells, and B cells. It was initially discovered through its role in preventing the production of T_H_1 cytokines in mice (Moore et al., 2001). It signals via the IL-10 receptor (IL-10R) to activate the JAK/STAT pathway and exerts immuno-suppressive effects by blocking IκK activity (Schottelius et al., 1999) or by inducing tyrosine phosphorylation of STAT-3 (Lumeng et al., 2007). Interleukin-10 may play a protective role in obesity-induced metabolic dysregulation and IR. Interleukin-10 levels are attenuated in T2D (Van Exel et al., 2002) and weight loss enhances WAT IL-10 expression coincident with reduced pro-inflammatory gene expression (Cancello et al., 2005). Lumeng et al. have demonstrated elevated IL-10 expression in ATM and within the SVF of lean WAT compared with obese WAT. Interleukin-10 reduced MCP-1 secretion from 3T3-L1-adiopcytes. Moreover pre-treatment of 3T3-L1-adiopcytes with IL-10 blocked the insulin-desensitizing effects of TNF-α and enhanced insulin-stimulated glucose transport (Lumeng et al., 2007). Interleukin-10 is associated with many immune cells and production by T_H_ cells initiates a feedback loop that can limit the effector functions of macrophages and DCs. It can also drive differentiation of IL-10 secreting T_reg_ cells (Saraiva and O’Garra, 2010). Interleukin-10 can inhibit the production of MHC class II and co-stimulatory molecule expression in DCs and macrophages, it can also prevent the production of cytokines from CD4^+^ T cells (Joss et al., 2000; Couper et al., 2008).

Other studies have challenged the protective effects of IL-10 in obesity-induced IR. Deletion of hematopoietic cell derived IL-10 did not exacerbate WAT or liver inflammation in response to HFD. However IL-10 expression was markedly up-regulated in WAT and liver in these mice compared to wild type (WT) mice, suggesting induction of a compensatory mechanism (Kowalski et al., 2011). Therefore further *in vivo* studies are required to evaluate the significance of IL-10 in obesity-induced IR.

### Interleukin-6

Interleukin-6 is secreted by WAT, skeletal muscle, and the liver (Fasshauer et al., 2003; Weisberg et al., 2003; Wieckowska et al., 2008). Interleukin-6 signals via the JAK/STAT and MAPK pathways (Heinrich et al., 2003). WAT and plasma IL-6 expression is correlated with BMI (Vozarova et al., 2001) and up-regulated by insulin and TNF-α; it negatively impacts on insulin signaling promoting serine phosphorylation of IRS-1 (Fasshauer et al., 2003; Ruge et al., 2009). Interleukin-6 may promote dysregulation of FA metabolism in WAT as it enhances mesenchymal stem cell proliferation, maintaining the cells in an undifferentiated state and inhibiting adipogenesis (Pricola et al., 2009). Increased hepatic IL-6 levels are also associated with steatohepatitis and plasma IL-6 levels (Wieckowska et al., 2008). Additionally IL-6 was recently shown to stimulate insulin secretion via enhanced GLP-1 expression in pancreatic cells (Ellingsgaard et al., 2011). This suggests that obesity-induced IL-6 secretion may reflect a mechanism to increase insulin production in the obese IR state. However, while elevated IL-6 secretion from WAT and the liver is unfavorable, the opposite is true for skeletal muscle. Physical inactivity decreases insulin sensitivity, it is also associated with reduced skeletal muscle IL-6 expression and secretion (Pedersen and Febbraio, 2012). Furthermore the increase in plasma IL-6 levels that result from exercise are followed by increased IL-1RA and IL-10 levels, and exercise induced IL-6 is thought to result from glycogen/MAPK activation rather than through NF-κB (Pedersen, 2011); thus highlighting the pleiotropic role of IL-6.

### Macrophage migration inhibitory factor

Several studies suggest an association between MIF and obesity-induced IR (Vozarova et al., 2002; Dandona et al., 2004; Ghanim et al., 2004; Church et al., 2005). Macrophage migration inhibitory factor has a prominent role in macrophage biology, it promotes secretion of TNF-α, IL-6, IL-1β, and inhibits IL-10, propagating a pro-inflammatory response (Calandra et al., 1994; Baugh and Bucala, 2002; Calandra and Roger, 2003; Lue et al., 2007). Moreover MIF can sustain activated macrophage life span by thwarting p53-dependent apoptosis (Mitchell et al., 2002) and induce chemotaxis via CXCR2 and CXCR4 in macrophages and T cells respectively (Bernhagen et al., 2007). Plasma MIF concentrations and PBMC MIF mRNA are positively associated with BMI, FFA concentration, impaired glucose tolerance (IGT), and IR (Vozarova et al., 2002; Skurk et al., 2005). Obese SAT explants demonstrate increased MIF secretion, and expression is increased with adipocyte size IR (Skurk et al., 2005; Koska et al., 2009). Weight loss and treatment with the anti-diabetic drug metformin reduces plasma MIF concentrations coincident with improved pancreatic β-cell function (Dandona et al., 2004; Church et al., 2005). More recently a causal role for MIF in IR was emphasized using an atherosclerotic mouse model which lacked functional MIF (LDLR^−/−^MIF^−/−^). This model displayed less local WAT inflammation, reduced MAC3^+^ macrophage infiltration, with enhanced WAT and systemic insulin sensitivity (Verschuren et al., 2009). In addition deletion of MIF reduced monocyte adhesion, macrophage lesion content, and atherosclerotic lesion size (Verschuren et al., 2009). Unpublished work within our group implicates MIF as a critical inflammatory regulator in the pathogenesis of HFD-induced IR with an essential role in HFD-associated ATM recruitment. It has since emerged that lack of MIF signaling results in an age-dependent impairment of glucose homeostasis in mice fed a chow diet (Serre-Beinier et al., 2010), highlighting the intricacy of this molecule and lack of true clarity in terms of its functional role in obesity-induced IR.

### Adiponectin

Adiponectin is a 30-kDa secretory hormone produced predominantly by adipocytes, expression, and secretion are elevated during adipocyte differentiation (Carbone et al., 2012). Reduced adiponectin levels such that accompany obesity, result in IGT due to reduced insulin sensitivity (Arita et al., 1999; Fantuzzi, 2005). There are three major isoforms of adiponectin; low molecular weight (LMW), formed from three adiponectin monomers, middle-molecular weight (MMW) which is an octomer, and high-molecular weight (HMW) consisting of 12 or more monomers (Magkos and Sidossis, 2007). HMW adiponectin is the most biologically active form and best reflective of the reduction in total adiponectin levels associated with obesity (Almeda-Valdes et al., 2010). Indeed HMW-adiponectin levels have been identified as an independent risk factor for IR (Aso et al., 2006; Hara et al., 2006; Almeda-Valdes et al., 2010).

Adiponectin can enhance FA oxidation and improve insulin sensitivity (Carbone et al., 2012); suppressing gluconeogenesis in the liver thus reducing circulating glucose levels, also increasing glucose uptake in the muscle by enhanced GLUT4 expression. In the liver, adiponectin stimulates glucose and FA oxidation through activation of the AMPK pathway and reduces lipogenesis by minimizing SREBP-1c expression (Utzschneider and Kahn, 2006).

In addition to its insulin sensitizing role, adiponectin is typically anti-inflammatory. Adiponectin can suppress the production of TNF-α and IFN-γ and it is speculated to be a negative regulator of T cells (Scherer et al., 1995; Carbone et al., 2012). Tsang et al. (2011) demonstrated that chronic adiponectin treatment in DCs decreased the expression of the co-stimulatory molecules CD80 and CD86, MHC II, and also IL-12. Additionally adiponectin treated DCs had altered T cell interactions with reduced proliferation of T_H_1 cells and T_reg_ cell induction. Thus targeting adiponectin signaling pathways may be promising in the treatment of metabolic diseases.

## Cytokine Synergy to Augment Obesity-Induced Insulin Resistance

Given the complexity of pro-inflammatory signaling, it is highly probable that a combination of stimuli rather than a single entity would elicit greater accountability for the inflammation-IR paradigm. Certainly it is known that IL-1, TNF-α, and TLR4 converge at the inhibitor of NF-κB kinase complex (IκB kinase) (Verstrepen et al., 2008). McGillicuddy et al. (2011) emphasized the potent synergistic capabilities IL-1β and TNF-α in macrophages and WAT. Intriguingly this study showed that lack of IL-1 signaling altered the immunogenic phenotype of ATM, with reduced IL-6 and TNF-α secretion, but without altered ATM number. Correspondingly AT inflammation was reduced concomitant with enhanced insulin-stimulated pAKT expression. This study also showed that adipocytes exposed to IL-1β and TNF-α induced greater activation of NF-κB, with significant amplification of IL-6 secretion compared with either cytokine alone. Furthermore abrogation of IL-1R1 in bone marrow macrophages ablates these synergistic abilities. To investigate this phenomenon in a more physiological relevant setting, WAT explants from WT and IL-1R1^−/−^ mice were cultured in the presence of IL-1β and TNF-α *ex vivo*, loss of synergy was again observed in IL-1R1^−/−^ WAT. Another important inflammatory regulator that offers potential involvement in IL-1, TNF-α, and TLR4 signaling is MIF (Finucane et al., 2012). Endogenous MIF regulates innate immunity via upregulation of TLR4, IL-1R1, and p55 tumor-necrosis factor receptor (TNFR) expression (Roger et al., 2001; Toh et al., 2006). MIF^−/−^ macrophages were shown to be hypo-responsive to LPS and failed to secrete TNF-α and IL-6 due to profound suppression of IKKβ/NF-κB activity (Roger et al., 2001). Macrophage migration inhibitory factor deficiency suppressed IL-1 and TNF-α induced MAPK activity and cell proliferation in mouse fibroblasts. Reconstitution of an upstream MAPK or MIF reversed this suppression (Toh et al., 2006). Furthermore, treatment of 3T3-L1 adipocytes with TNF-α induced MIF secretion, suggesting that TNF-α may regulate MIF production during obesity (Hirokawa et al., 1997, 1998). *In vivo* MIF deficient mice were hypo-responsive to TNF-α and exhibited euglycemia, indicating MIF is required for successful TNF-α action. Additionally 3T3-L1 adipocytes exposed to exogenous MIF demonstrated impaired insulin-stimulated glucose uptake and insulin receptor signal transduction. Similarly in response to inflammatory stress MIF^−/−^ mice exhibit a marked improvement in WAT glucose uptake compared to control mice (Atsumi et al., 2007).

## Activation and Propagation of Inflammation and Insulin Resistance

**Figure 4:**
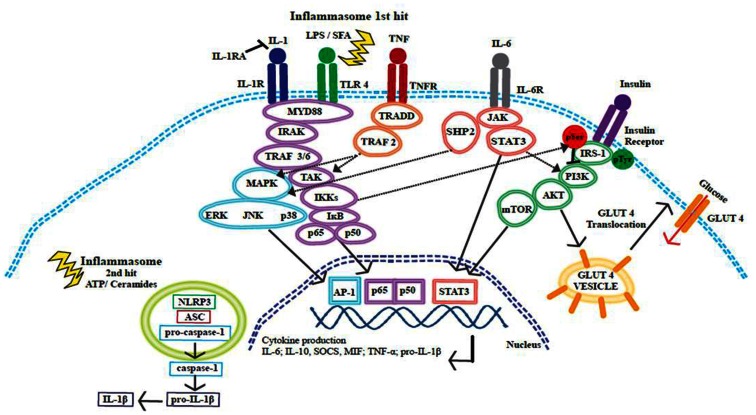
**Cross-talk between insulin and inflammatory signaling pathways**. Inflammatory signaling pathways activated by SFA or by pro-inflammatory cytokines IL-1β, IL-6, and TNF-α initiate a cascade of events that promote the release of further inflammatory mediators. These signaling events converge at the NF-κB and MAPK pathways, resulting in the translocation of transcription factors to the nucleus, transcriptional activation, and cytokine production. The inflammasome is activated through a two-hit process; with obesity the first hit occurs when TLR4 is activated by SFAs and this results in pro-IL-1β production, ATP or ceramides then provide the second hit. The NLRP3 inflammasome acts on pro-caspase-1 causing the release of caspase-1; caspase-1 then acts upon pro-IL-1β cleaving this precursor to the active IL-1β form. Insulin signaling promotes glucose uptake by promoting the translocation of GLUT4 to the cell surface plasma membrane. Inflammatory signaling pathways can alter the phosphorylation status of IRS-1. IRS-1 is crucial in the insulin signaling pathway, tyrosine phosphorylation is associated with an insulin sensitive state. IKKβ and JNK can promote serine phosphorylation of IRS-1 and this phosphorylation state is linked to insulin resistance and reduced glucose uptake.

**Figure 5:**
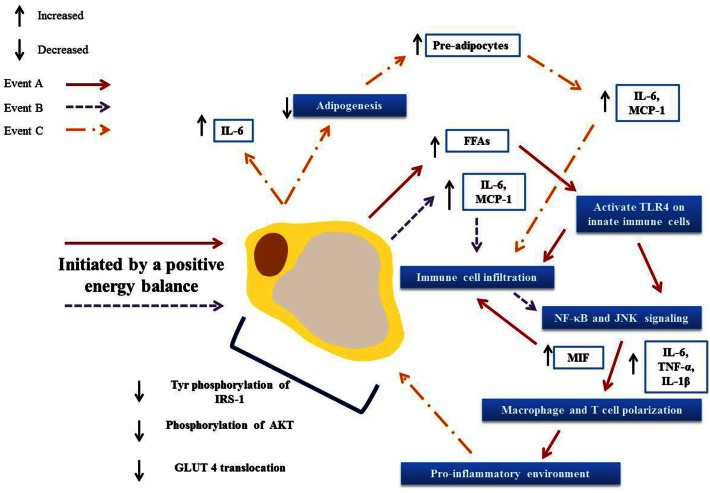
**Activation and propagation of inflammation and insulin resistance in obese adipose tissue**. Adipocyte hypertrophy results in elevated circulation of FFAs **(A)** and increased secretion of adipokines **(B)**. These in turn result in immune cell infiltration and the activation of pro-inflammatory signaling pathways, driving further infiltration and the polarization of adipose tissue macrophages and T cells toward a pro-inflammatory phenotype. This environment then drives further adipokine secretions **(C)** and hampers adipogenesis, resulting in greater numbers of pre-adipocytes that in turn secrete pro-inflammatory mediators. Together these events lead to defective insulin signaling and ultimately to an insulin resistant state.

The NF-κB and JNK MAPK pathways link obesity, inflammation, and IR, summarized in Figure 4 (Hirosumi et al., 2002; Shoelson et al., 2003). But despite intense scrutiny, an intricate question persistently evades resolution, what instigates and consequentially propagates this inflammation? See Figure 5. Reduced ability of WAT to adequately store excess fat and an increased hypoxic environment have been proposed as two potential contributors. Hypoxia represents a metabolic stressor relevant to obese WAT, this local hypoxia may be partially attributed to an insufficient blood supply reaching the expanding organ (Kabon et al., 2004; Hosogai et al., 2007). Hypoxia-inducible factor-1 (HIF)-1 is a transcription factor with an oxygen regulated α subunit (HIF-1 α) (Bruning et al., 2011; Shin et al., 2012); tissue inflammation is associated with regions of hypoxia, and HIF signaling is also linked to NF-κB activation (Ye et al., 2007; Bruning et al., 2012). WAT hypoxia correlated with reduced expression of adiponectin in a HFD-fed mouse model. Moreover, hypoxic conditions augment WAT derived cytokines including leptin and IL-6 (Sun et al., 2011). Conversely, weight loss improves adipose oxygenation and increases adiponectin expression. HIF-1α is a critical regulator of cellular hypoxic responses and is rapidly degraded under normoxic conditions. HIF-1α activity increases early in obesity. Overexpression of HIF-1α initiates WAT fibrosis, accompanied by increased local inflammation. Halberg et al. (2009) suggest that hypoxia induced WAT fibrosis may be responsible for initiating WAT inflammation during obesity. A HIF-1α antisense oligonucleotide (ASO) suppressed *Hif-1*α gene expression in the liver and WAT of HFD-fed mice (Shin et al., 2012), and resulted in weight loss without a change in food intake or activity levels. *In vitro* analysis of hypoxia in tumor cell lines showed that resveratrol (Zhang et al., 2005; Park et al., 2007) and two isoforms of conjugated linoleic acid (CLA) *c*9,*t*11- and *t*10,*c*12-CLA (Yamasaki et al., 2012) can promote HIF-1α protein degradation; suggesting that the hypoxia related inflammatory response in WAT is nutrient sensitive.

Decreased WAT expandability may result in lipid spillover, elevating circulating FFAs resulting in enhanced ectopic lipid accumulation in peripheral tissues (Khan et al., 2009; Prieur et al., 2011). Long-chain (LC) SFA can serve as a ligand to the innate immune receptor TLR4 (Shi et al., 2006). Indeed, exposing macrophages and adipocytes to varying concentrations of palmitate, the most abundant circulating SFA in obesity, elicits a TLR4 dependent pro-inflammatory response characterized by increased NF-κB and JNK activation with resultant enhanced TNF-α cytokine secretion *in vitro*. Furthermore TLR4^−/−^ mice administered an acute lipid infusion maintained glucose homeostasis (Shi et al., 2006). HFD-fed mice lacking TLR4 had attenuated IR, increased energy expenditure and improved WAT inflammation (Shi et al., 2006; Tsukumo et al., 2007). While bone-marrow transplant studies indicate that TLR4 in the hematopoietic system opposed to the non-hematopoietic system may be responsible for TLR4 mediated IR during HFD wherein TLR4 chimeric mice exhibit reduced hyperinsulinemia, hepatic, and WAT IR (Saberi et al., 2009). Despite abundant reports signifying that TLR4 ameliorates WAT inflammation, the effect on ATM accumulation has remained unclear. In this regards Orr et al. (2012) demonstrated TLR4 deficiency did not alter ATM number but promoted ATM toward an M2 anti-inflammatory phenotype.

Dietary FA have been shown to interact with DCs and alter cell function (Loscher et al., 2005; Weatherill et al., 2005; Zeyda et al., 2005). Saturated fatty acid drives DC maturation through interaction with TLR4. Bone marrow derived dendritic cells (BMDCs) isolated from HFD-fed mice are skewed toward a pro-inflammatory phenotype with increased secretion of IL-1β, IL-12p70, and TNF-α in response to LPS (Reynolds et al., 2012). Interleukin-1R1, TLR4, caspase-1, and NLRP3 mRNA expression also increases in BMDCs from HFD-fed mice; additionally cell surface TLR4 expression was also significantly increased. Polyunsaturated fatty acids (PUFA) have an opposing effect on DC function and can suppress IL-12p70 production while boosting IL-10 release, thus driving a T_reg_ rather than a T_H_1 response (Loscher et al., 2005; Weatherill et al., 2005). PUFA largely exert an effect via changes in gene expression causing activation of transcription factors (Bouwens et al., 2010).

Specific FAs as well as some eicosanoids have been shown to bind to receptors of the PPAR family of transcription factors, including the PPAR-α, PPAR-β/δ, and PPAR-γ isoforms (Forman et al., 1997; Delerive et al., 1999). PPAR act as a hub between inflammation and insulin sensitivity, via physical interactions with NF-κB and IκBα (Ricote et al., 1998; Delerive et al., 2002); antagonizing inflammation and promoting insulin signaling. Activation of PPAR-γ is associated with the upregulation of IRS proteins (Hammarstedt and Smith, 2003; Seto-Young et al., 2007); additionally the thiazolidinedione class of drugs which act as PPAR-agonists, promote tyrosine phosphorylation of IRS-1 in both *in vivo* and *in vitro* models (Jiang et al., 2002, 2004). Nevertheless it is impossible to adequately address PPAR functionality in obesity within the current review and this topic was reviewed very comprehensively elsewhere (Stienstra et al., 2007; Varga et al., 2011).

Ectopic accumulation of excessive palmitate-derived lipid plays an antagonistic role in insulin signaling (Gill and Sattar, 2009). Ceramides in particular are postulated to be a critical factor connecting disproportionate lipid accretion, inflammation, and impaired insulin sensitivity (Summers, 2006). Indeed activation of a TLR4 inflammatory response via SFA is essential for SFA induced ceramide biosynthesis (Holland et al., 2011). Ceramides comprise of sphingosine bound to a FA and serve as both structural and signaling molecules (Gill and Sattar, 2009). Ceramides are generated by condensation of palmitoyl-CoA and serine via the rate-limiting enzymatic reaction mediated by serine palmitoyltransferase (Mathias et al., 1998; Summers, 2006). Importantly pro-inflammatory cytokine such as IL-1β amplify ceramide biosynthesis by upregulating serine palmitoyltransferase (Summers, 2006). Haus et al. (2009) ascribed a pathogenic association between total plasma ceramide and its subspecies C18:0, C20:0, C24:1, C24:0 concentrations and the degree of IR in obese T2D patients. Elevated circulating TNF-α levels were positively correlated with increased C18:1 and C18:0 ceramide subspecies.

Culturing C2C12 myoblasts in the presence of palmitate induced *de novo* ceramide accumulation, disrupting insulin-stimulated pAKT activation. Inhibiting ceramide accumulation negated the detrimental effects on insulin signaling. However, whether ceramides induced an inflammatory response in myoblasts was not assessed in this study (Chavez et al., 2003). Further, Holland et al. demonstrated enhanced ceramide accumulation in the soleus muscle in response to lard oil infusion but not soy oil infusion, a source of PUFAs. Inhibiting ceramide infusion using serine palmitoyltransferase inhibitors prevented ceramide induced IR. Additionally lard oil evoked a significant increase in circulating pro-inflammatory IL-6 and TNF-α (Holland et al., 2011). Most recently ceramides were identified as an effective second “hit” required for NLRP3 inflammasome activation in macrophages. Furthermore, ceramides evoked NLRP3 dependent caspase-1 activation in obese WAT explants, an event that was lost in NLRP3^−/−^ WAT (Vandanmagsar et al., 2011). Despite significant evidence highlighting the importance of ceramides in inflammation and IR the clinical importance of these findings is yet to be fully deciphered. A subsequent study reported that palmitate was a potential second signal required for NLRP3 activation, inducing IL-1β production from macrophages. Both NLRP3 and the inflammasome adaptor ASC (apoptotic speck-like protein containing a CARD, known also as Pycard) deficiency ameliorated obesity-induced IR in mice (Wen et al., 2011).

## Insulin Resistance as a Protective Mechanism

It has been hypothesized that the induction of IR is perhaps a protective response. Insulin resistance could potentially act to protect cells from stress and damage through the exclusion of glucose from cells that are heavily lipid loaded; thus reducing the chance of lipotoxic damage (Unger, 2003). *In vivo* and *in vitro* rat studies indicate that glucose toxicity occurs when cells are exposed to a chronic hyperglycemic state (Rossetti et al., 1990), and this may induce or aggravate IR (Baron et al., 1995). Impaired GLUT 4 translocation in response to the metabolite glucosamine has been implicated in the induction of IR (Baron et al., 1995). Roden et al. (1996) determined through lipid infusion studies in healthy volunteers, that FFA’s inhibit glucose transport. It has since been demonstrated that prolonged FFA exposure but not short-term exposure, interrupts glucose uptake (Hawkins et al., 1997). Alternatively IR may be induced partly in response to oxidative stress. Oxidative stress describes a biological state that develops following increased ROS production or reduced ROS clearance (Azzi, 2007). ROS production increases in response to increased macronutrient consumption (Codoñer-Franch et al., 2011). Excessive nutrient intake is linked to an increase in superoxide (O_2_^•^−) generation by mitochondria (Tiganis, 2011). Mitochondria produce most of the cell’s energy, and in this capacity take up the majority of intracellular oxygen (Finkel and Holbrook, 2000). Cell signaling pathways are activated in response to increasing oxidative stress; these pathways include MAPK, NF-κB, and the PI3K-AKT pathway (Finkel and Holbrook, 2000). Hoehn et al. (2009) propose that IR is linked to mitochondrial O_2_^•^− and that O_2_^•^− acts as a nutrient sensor, which regulates nutrient intake under conditions of overnutrition. This group reports that mitochondrial O_2_^•^− is upstream of IR in skeletal muscle and AT; overexpression of mitochondrial superoxide dismutase (MnSOD) enzymes, with anti-oxidant action, in *in vitro* and *in vivo* rodent models significantly improved insulin sensitivity. Additionally, findings from caloric restriction (CR) studies show reduced oxidative damage (Sohal et al., 1994; Sohal and Weindruch, 1996; Masoro, 2000; Zainal et al., 2000). Sirtuin 1 (SIRT1) a member of the sirtuin family of proteins, may mediate the effect of CR by influencing PPAR-γ, PGC1-α, and FOXO (Han et al., 2010). SIRT1 has previously been shown to improve insulin sensitivity in *in vitro* and *in vivo* mouse studies (Moynihan et al., 2005; Sun et al., 2007).

## Metabolically Healthy Obese

Interestingly 20–30% of obese adults do not express the adverse metabolic phenotype typically associated with obesity (Alam et al., 2012). Individuals considered metabolically healthy but obese (MHO) have high levels of insulin sensitivity but may not display symptoms of hypertension, dyslipidemia, or chronic inflammation (Karelis et al., 2005; Stefan et al., 2008; Succurro et al., 2008). MHO individuals have significantly smaller omental adipocytes than metabolically unhealthy individuals (O’Connell et al., 2010). This finding correlates with the degree of IR and hepatic steatosis within the obese groups. Interestingly this study demonstrated that BMI was not associated with adipocyte size nor did it predict general metabolic health or fatty liver disease, in a group of adults with a median BMI of 48 kg/m^2^. Brochu et al. (2001) demonstrated in a group of obese postmenopausal women, that MHO individuals had lower levels of VAT; and that these women had been obese for longer than the metabolically unhealthy group. Additionally the MHO group was shown to have lower plasma TAG levels and higher high-density lipoprotein (HDL) cholesterol concentrations. In a study of obese males and females, fitness levels were higher in the MHO group (Ortega et al., 2013). Conversely an individual may be considered metabolically obese but normal weight (MONW) (Ruderman et al., 1981). Adult female subjects determined to be MONW tend to have a similar BMI to their metabolically healthy counterparts but have a higher percentage body fat, lower fat-free mass, and lower physical activity energy expenditure, and are considered to be less aerobically fit (Conus et al., 2004). Female MONW subjects were shown to have increased plasma cholesterol and reduced insulin sensitivity but showed no change in ghrelin, leptin, or adiponectin levels (Conus et al., 2004). MONW subjects also have increased TAG and FFA levels. Low-HDL cholesterol levels have been noted in male MONW subjects when compared with healthy non-obese subjects (Succurro et al., 2008; Lee et al., 2011).

## Targeting Nutrient-Sensitive Inflammatory Pathways to Treat Insulin Resistance

There is no doubt that weight loss can improve the inflammatory phenotype and insulin sensitivity (Kopp et al., 2005; Kováciková et al., 2011). However, maintenance of weight loss is difficult to achieve and weight regain frequently occurs (Gage, 2012). Thus there is a vital need to identify alternative nutritional interventions that may antagonize inflammation, independent of weight loss. As proof of concept from a pharmaceutical perspective, drugs that (1) interfere with the TLR4/IKK/NF-kB axis, (2) target PPAR-γ, or (3) target pro-inflammatory cytokines, have demonstrated promise with respect to treating IR despite obesity. Salicylate, an inhibitor of IKK has been shown to reverse hyperglycemia, hyperinsulinemia, and dyslipidemia in obese rodents by sensitizing insulin signaling (Yuan et al., 2001). Anakinra, an IL-1 RA, was shown to improve glycemia and beta-cell secretory function in patients with T2D (Malozowski et al., 2007). Small molecule MIF antagonist CPSI-1306 treatment in a mouse model with streptozotocin (STZ)-induced T2D, resulted in reduced circulating IL-6 and TNF-α levels and reduced blood glucose levels (Sanchez-Zamora et al., 2010). Nevertheless, long-term pharmacological immuno-suppressive interventions that attenuate IR, may not be ideal in terms of immunosurveillance and long-term health (Kung and Henry, 2012; Bortolini et al., 2013). Alternatively, anti-inflammatory nutritional interventions may have potential. Albeit producing a more subtle effect, several nutrients have now emerged as potentially insulin sensitizing, affecting the same molecular targets as established pharmaceutical approaches.

Perhaps the most extensively researched in relation to their immunomodulating effects are dietary FA which are known to interact with several inflammatory pathways (Hotamisligil and Erbay, 2008). The effect of FAs on these pathways depends on the degree of FA saturation (Bradley et al., 2008). Within this context, eicosapentaenoic acid (EPA) and docosahexaenoic acid (DHA), two LC *n* − 3 PUFAs have emerged as anti-inflammatory nutrients that exert their effects through a number of biological mechanisms. Opposingly, SFA activate TLR4 and increase NF-κB transcriptional activity. EPA and DHA may mitigate this response (Bradley et al., 2008; Reynolds et al., 2012) by reducing nuclear p65 expression and increasing cytoplasmic IκBα expression, and DHA may act as a more potent NF-κB inhibitor than EPA (Weldon et al., 2007). Xue et al. (2012) suggested that this effect is partially mediated via AMPK/SIRT1 activation as DHA does not fully deacetylate p65 in SIRT1 knockdown macrophages. Furthermore pre-treatment of macrophages with DHA promotes an anti-inflammatory phenotype, with reduced IL-6 and increased IL-10 expression; which when co-cultured with adipocytes attenuates the characteristic IR phenotype (Oliver et al., 2012). Therefore the anti-inflammatory effects observed due to DHA pre-treatment were translated into improved insulin sensitivity in adipocytes (Oliver et al., 2012).

The GPCR GPR120 that is highly expressed in both adipocytes and macrophages, plays a pivotal role in LC *n* − 3 PUFA mediated inhibition of inflammation and IR (Oh et al., 2010). Both EPA and DHA bind to the GPR120 receptor to inhibit NF-κB and JNK via reduced TAK1 phosphorylation (Oh et al., 2010). Furthermore it was demonstrated that LC *n* − 3 PUFA supplementation increased insulin sensitivity in WT but not in GPR120^−/−^ mice (Oh et al., 2010). Additionally LC *n* − 3 PUFA may exert their anti-inflammatory effect by enhancing adiponectin secretion from human adipocytes, an effect that is elicited at least partially, via PPAR-γ (Tishinsky et al., 2011). These results are consistent with *in vivo* work that demonstrated feeding mice a fish oil enriched diet increases plasma adiponectin, and this was completely blocked by the PPAR-γ antagonist BADGE (Neschen et al., 2006).

Long-chain *n* − 3 PUFA may increase β-oxidation in WAT and cultured adipocytes (Guo et al., 2005; Flachs et al., 2011), potentially via activation of the AMPK regulatory pathway (Lorente-Cebrián et al., 2009; Figueras et al., 2011). This metabolic switch could increase the mitochondrial content of adipocytes, resulting in reduced accumulation of toxic FA derivatives and improved insulin sensitivity (Kopecky et al., 2009). Furthermore, LC *n* − 3 PUFA competitively inhibit the conversion of arachidonic acid (AA) to pro-inflammatory eicosanoids such as prostaglandin E2 and leukotriene B4. Increased intake of LC *n* − 3 PUFA promote the incorporation of EPA into membrane phospholipids at the expense of AA, thereby increasing production of EPA-derived anti-inflammatory eicosanoids, such as prostaglandin E3 and leukotriene B5 (Lottenberg et al., 2012). Whilst the potential benefits of LC *n* − 3 PUFA supplementation on inflammatory pathways is clear *in vitro*, it is difficult to ascertain a consistent effect in man (Kabir et al., 2007; Tierney et al., 2011). Inconsistencies in human data are reflective of dose variability between studies, duration of supplementation, and the population studied (Calder et al., 2011). Genetic variability between individuals likely influences responsiveness to an intervention (Calder et al., 2011). Single nucleotide polymorphisms (SNPs) can influence responsiveness, MetS patients that are minor allele carriers of an adiponectin SNP have reduced IR following reduced SFA intake (Ferguson et al., 2010). A second study showed that common genetic variants of the complement component 3 (C3) locus conferred an increased risk of MetS, and that PUFA intake may modulate these genetic influences (Phillips et al., 2009a). Therefore gene-nutrient interactions may play an important role in regards to responsiveness to interventions, a personalized nutrition approach may be considered in order to determine an ideal dietary intervention.

There is growing evidence, albeit highly controversial, in relation to the immunomodulating potential of vitamin D. Following *in vitro* induced inflammation, 1,25-dihydroxyvitamin D up-regulates IκBα in macrophages through increased mRNA stability and decreased IκBα phosphorylation, thus reducing NF-κB activity (Cohen-Lahav et al., 2006). Furthermore, 1,25-dihydroxyvitamin D suppresses TLR2 and TLR4 expression in human monocytes (Sadeghi et al., 2006). Du et al. (2009) demonstrated vitamin D_3_ pre-treatment of monocytes from T2D patients and controls resulted in similar TLR2 and TLR4 expression, NF-κB p65 phosphorylation state, and IL-1β and TNF-α expression in the two groups. Nevertheless human data is inconsistent, intervention studies have shown little or no effect of vitamin D supplementation on inflammatory or metabolic markers related to insulin sensitivity (Gulseth et al., 2010; O’Sullivan et al., 2011). Furthermore cross-sectional data showed that serum vitamin D status bore no relationship with insulin action or secretion in subjects with the MetS (Gulseth et al., 2010).

Vitamin C and vitamin E have been proposed to improve insulin sensitivity through anti-oxidant and anti-inflammatory mechanisms. As an anti-oxidant, ascorbic acid down-regulates ROS that otherwise would lead to activation of NF-κB (Nathan, 2003). Moreover, after oxidation to dehydroascorbic acid, vitamin C has been shown to directly inhibit IKKα, IKKβ, and p38 MAPK activity (Cárcamo et al., 2004). Vitamin C has also been shown to inhibit nitric oxide (NO) production and decrease insulin-induced MCP-1 and apelin secretion in an adipocyte-macrophage co-culture (Garcia-Diaz et al., 2011). Similarly, the insulin sensitizing effect of lycopene, a carotenoid pigment found in tomatoes involves inhibition of NF-κB, NO, and IL-6, and suppresses the activation of a number of MAP kinases (Feng et al., 2010). Phosphorylation of the MAP kinases ERK, p-38, and JNK, were shown to be ameliorated by lycopene (Kim et al., 2004). In WAT, pro-inflammatory cytokine and chemokine expression were reduced by lycopene treatment (Gouranton et al., 2011). A recent study conducted in young overweight adults showed that daily supplementation with one glass of tomato juice reduced TNF-α, IL-6, and IL-8 after 20 days (Ghavipour et al., 2012). Another study in overweight men demonstrated a decrease in systemic levels of serum amyloid A after 12 weeks of lycopene supplementation (McEneny et al., 2012). However Thies et al. (2012) demonstrated that following a 12-week treatment on a tomato-rich diet, inflammatory markers such as highly sensitive CRP (hsCRP) and IL-6, and HOMA-IR, remained unchanged.

Polyphenols, particularly flavonoids are gaining increasing attention for their anti-inflammatory effect. Resveratrol, a polyphenol that naturally occurs in grapes (Dong, 2003) was shown to inhibit pre-adipocyte proliferation, adipogenic differentiation, and *de novo* lipogenesis in a SIRT1-dependent manner in SGBS (Simpson–Golabi–Behmel syndrome) adipocytes (Fischer-Posovszky et al., 2010). Interestingly in humans it has been demonstrated that resveratrol treatment can improve the metabolic phenotype of healthy obese men, by reducing blood glucose and insulin levels and reducing plasma inflammatory markers (Timmers et al., 2011). Nevertheless it must be acknowledged that there have been other resveratrol interventions which have had little effect (Poulsen et al., 2012; Yoshino et al., 2012). Poulsen et al. demonstrated that resveratrol failed to improve endogenous glucose production and turnover or improve inflammatory and metabolic biomarkers in obese but otherwise healthy men. Additionally plasma lipids and insulin sensitivity did not improve; neither was there a change in molecular targets such as AMPK or SIRT1 in non-obese women treated with resveratrol (Yoshino et al., 2012).

Epigallocatechin gallate (EGCG) present in green tea, has been implicated in inhibiting resistin gene expression in adipocytes (Liu et al., 2006) and was shown to decrease ERK phosphorylation. Yang et al. (2001) demonstrated EGCG also inhibits IKK. A recent investigation of quercetin, demonstrated that this flavonoid, found predominantly in capers, apples, and grapes, attenuated TNF-α-induced NF-κB activity and subsequent expression of inflammatory genes in primary human adipocytes (Chuang et al., 2010).

Curcumin which is the yellow pigment found in the spice turmeric inhibits LPS-induced secretion of TNF-α and IL-1β *in vitro* (Chan, 1995). Curcumin also completely inhibits TNF-α-induced activation of NF-κB and other inflammatory agents such as phorbol esters (Singh and Aggarwal, 1995). Salicylic acid, the basic component of aspirin is present in fruits, vegetables, herbs, and spices (Duthie and Wood, 2011). Relatively low concentrations of salicylic acid have been shown to inhibit cyclooxygenase (COX)-2 *in vitro* (Wu et al., 1991; Xu et al., 1999; Hare et al., 2003) and suppress the transcriptional activation of pro-inflammatory genes such as iNOS (Duthie and Wood, 2011). However, salicylate concentrations sufficient to inhibit NF-κB activation are unlikely to be achieved through diet, limiting the therapeutic potential of salicylic acid in its natural form (Duthie and Wood, 2011; Wood et al., 2011).

Much of the research to date has examined the effect of individual nutrients on inflammation however recent evidence suggests that a combination of nutrients may offer an enhanced immunomodulating effect. Bakker et al. (2010) demonstrated that supplementing overweight men with a combination of nutrients, with known anti-inflammatory properties, increased plasma adiponectin by 7% over a 5-week period, independent of weight loss. Additionally, large-scale profiling of genes, proteins, and metabolites showed that the intervention could influence inflammation, oxidative stress, and metabolism. Anti-inflammatory IL-10Rα and SOCS3 expression were up-regulated in WAT in response to the intervention (Bakker et al., 2010). This study highlights the potential efficacy of nutritional interventions that target multiple signaling pathways to treat IR in obese individuals.

Despite convincing evidence from *in vitro* and animal studies to support the therapeutic potential of several nutrients within the context of obesity-induced inflammation and IR, results from clinical studies are not entirely consistent. Even high dose nutritional supplementation often fails to elicit an anti-inflammatory effect (Blok et al., 1997; Jellema et al., 2004; Pot et al., 2009). Much of the discrepancy observed may stem from the examination of supraphysiological doses in *in vitro* studies (Calder et al., 2011). Taken together a cocktail of nutrients may be more beneficial in ameliorating the inflammatory phenotype observed in a number of pathologies. There is no doubt that there is potential for nutritional anti-inflammatory agents to improve IR within the obese phenotype. Nevertheless demonstrating efficacy is confounded by defining an effective dose of individual dietary elements and/or defining potential synergies between anti-inflammatory nutrients/food components. Determining effective doses may be facilitated by the development of specific functional foods, enriched with the active agents. There is strong evidence that some individuals with the MetS who have a pro-inflammatory status due to their genotype and/or inflammatory phenotype may be more susceptible to the pro-inflammatory effect of dietary SFA (Phillips et al., 2009b, 2013). Furthermore their pro-inflammatory genotype/phenotype may determine potential responsiveness to an intervention. Therefore there may be a difference in efficacy between sub-cohorts of the obese population to anti-inflammatory interventions; and a dual approach in terms of removing SFA and augmenting nutritional anti-inflammatory agents may be required to achieve the desired effect to attenuate IR.

## Conclusion

Inflammation is a critical mediator in obesity-induced IR. WAT expansion and the influx of immune cells initiate a cascade of inflammatory events that directly contribute to defective insulin signaling and glucose uptake, resulting in systemic IR. The mechanisms driving the pathogenic environment of obese WAT are complex and not fully elucidated; evidence implicates that a combination of events converge, and escalate the pro-inflammatory state. Research suggests that nutritional anti-inflammatory interventions may attenuate IR, independent of weight loss. However, results from human studies so far remain inconsistent. Moving forward, greater consideration should be given to designing nutritional interventions that (1) target multiple signaling pathways and (2) take account of genetic polymorphisms in order to improve efficacy.

## Conflict of Interest Statement

The authors declare that the research was conducted in the absence of any commercial or financial relationships that could be construed as a potential conflict of interest.

## Abbreviations

AA, arachidonic acid; ACC, acetyl-CoA carboxylase; AKT, protein kinase B; AMPK, adenosine monophosphate activated protein kinase; AP-1, activator protein-1; APC, antigen presenting cell; ASC, adipocyte stem cells; ASC, apoptotic speck-like protein containing a CARD; ASO, antisense oligonucleotide; AT, adipose tissue; ATM, adipose tissue macrophage; ATP, adenosine triphosphate; BAT, brown adipose tissue; bFGF, basic fibroblast growth factor; BMDC, bone marrow derived dendritic cell; BMI, body mass index; C/EBP, CCAAT/enhancer binding protein; C3, complement component 3; CLA, conjugated linoleic acid; CLS, crown-like structures; COX, cyclooxygenase; CVD, cardiovascular disease; DAG, diacylglycerol; DC, dendritic cell; DHA, docosahexaenoic acid; DPP, dipeptidyl peptidase; EGCG, epigallocatechin gallate; EPA, eicosapentaenoic acid; ERK, extracellular signal-regulated kinase; FA, fatty acid; FFA, free fatty acid; G6P, glucose 6-phosphatase; GLP, glucagon-like peptide; GLUT, glucose transporter; GPCR, G protein-coupled receptor; HDL, high-density lipoprotein; HFD, high-fat diet; HIF, hypoxia-inducible factor; HMW, high-molecular weight; HSD1, hydroxysteroid dehydrogenase type 1; IFN, interferon; IGT, impaired glucose tolerance; IκB, inhibitor of NF-κB; IKK, IκB kinase; IL, interleukin; iNKT, invariant natural killer cell; iNOS, inducible nitric oxide synthase; IR, insulin resistance; IRAK, interleukin-1 receptor-associated kinase-1; IRS, insulin receptor substrate; IκB, inhibitor of NF-κB kinase complex; JAK, janus kinase; JNK, Jun N-terminal kinase; KC, Kupffer cell; LC, long chain; LDL, low-density lipoprotein; LMW, low molecular weight; LPS, lipopolysaccharide; MAPK, mitogen activated protein kinase; MCP, macrophage chemoattractant protein; MDMs monocyte derived macrophages; MetS, metabolic syndrome; MHO, metabolically healthy obese; MIF, macrophage migration inhibitory factor; MMW, middle-molecular weight; MnSOD, mitochondrial dismutase; MONW, metabolically obese normal weight; mTOR, mammalian target of rapamycin; MYD88, myeloid differentiation primary response gene 88; NAFLD, non-alcoholic fatty liver disease; NK, natural killer cell; NLRP3, NOD, like receptor 3; NO, nitric oxide; O_2_^•^− superoxide; PI, phosphatidylinositol; PI3K, phosphatidylinositide 3-kinase; PKC, protein kinase C; PLC, phospholipase; PPAR, peroxisome proliferator-activated receptor; Pref-1, Pre-adipocyte factor-1; PRR, pattern recognition receptor; PUFA, polyunsaturated fatty acid; RA, receptor antagonist; ROS, reactive oxygen species; SAT, subcutaneous adipose tissue; SFA, saturated fatty acid; SGBS, Simpson–Gibson–Behmel syndrome; SHP2, protein tyrosine phosphatase, non-receptor type 11; SIRT1, sirtuin1; SNP, single nucleotide polymorphism; SOCS, suppressor of cytokine signaling; SREBP, sterol receptor binding protein; STAT, signal-transducer and activator of transcription protein; SVF, stroma-vascular fraction; T2D, type 2 diabetes; TAG, triacylglyceride; TAK, transforming growth factor β activated kinase; TH, T helper cell; TLR, toll like receptor; TNF, tumor-necrosis factor; TRADD, TNF receptor death domain; TRAF, TNF receptor-associated factor; Treg, regulatory T cell; VAT, visceral adipose tissue; WAT, white adipose tissue; WHO, world health organization; WT, wild type.
